# Production of Prodigiosin Using Tannery Fleshing and Evaluating Its Pharmacological Effects

**DOI:** 10.1155/2014/290327

**Published:** 2014-01-23

**Authors:** C. Sumathi, D. MohanaPriya, S. Swarnalatha, M. G. Dinesh, G. Sekaran

**Affiliations:** ^1^Council of Scientific and Industrial Research (CSIR), Central Leather Research Institute (CLRI), Adyar, Chennai, Tamil Nadu 600020, India; ^2^Saveetha Dental College and Hospitals, Chennai, India; ^3^Tamil Nadu Veterinary and Animal Sciences University, Chennai, India; ^4^National Institute of Environmental Research (Ministry of Environment), Incheon, Republic of Korea; ^5^Sri Ramachandra University, Chennai, India

## Abstract

*Aim*. The focal theme of present investigation includes isolation of prodigiosin producing fish gut bacteria, enhancing its production using tannery solid waste fleshing, and evaluation of its pharmacological effect. *Methods*. Optimization of fermentation conditions to yield maximum prodigiosin, and instrumental analysis using FTIR, NMR, ESI-MS, TGA, and DSC. *Results*. The optimum conditions required for the maximum prodigiosin concentration were achieved at time 30 h, temperature 30°C, pH 8, and 3% substrate concentration. The secondary metabolite was analyzed using ESI-MS, FTIR, and NMR. Therapeutic efficacy was assessed by in vitro anticancer studies. Among the pathogenic bacteria *Pseudomonas aeruginosa* was most susceptible at the lowest concentration followed by *Salmonella* typhi. IC_50_ concentration was cell line specific (HeLa cells: 4.3 *µ*M, HEp2: 5.2 *µ*M, and KB cells: 4.8 *µ*M) and remains nontoxic up to the concentration of 25 *µ*M on normal Vero cells suggesting that cancerous cells are more susceptible to the prodigiosin at lower concentration. *Conclusion*. Maximum prodigiosin production was obtained with tannery fleshing. The potency of the fish gut bacterial secondary metabolite prodigiosin as a therapeutic agent was confirmed through in vitro antimicrobial and anticancer studies.

## 1. Introduction

The production of clinically important microbial pigments is one of the emerging fields of research [1]. Prodigiosin is the major red pigmented secondary metabolite produced by *Serratia marcescens* [2] and several other bacteria such as *Vibrio psychroerythrus, Streptomyces coelicolor *A3*, Pseudomonas magnesiorubra, Streptomyces lividans, Alteromonas rubra, Streptomyces *spp.,* Hahella chejuensis *KCTC 2396*, Pseudovibrio denitrificans, Pseudoalteromonas rubra, *and* Nocardia* spp. [3, 4]. Intestinal bacteria have the capacity to hydrolyze the protein through fermentation and protect the host against diseases by producing secondary metabolites [5]. Hence, the secondary metabolite producing proteolytic bacteria from *Labeo rohita* gut was utilized for the fermentation of tannery fleshing to yield prodigiosin and its antagonistic activity against invading pathogens and abnormal cells were studied in detail. This is the first and foremost report on the production of prodigiosin from tannery solid waste fleshing.

## 2. Materials and Methods 

### 2.1. Effect of Various Parameters on Prodigiosin Production

The minimal medium containing different concentrations of carbon sources and nitrogen sources were initially used to optimize the effect of carbon and nitrogen source on growth of the bacteria and prodigiosin production at 37°C and the pH was maintained at 6.8. Using the optimized carbon and nitrogen sources, the physical parameters like pH, inoculum concentration, inoculum age, agitation rate, aeration rate, and substrate concentration were then optimized for maximum prodigiosin production. The optimization process was carried out changing one parameter at a time. Tannery fleshing (TF) was used as both the carbon and nitrogen source in the optimized condition for maximum prodigiosin production. All the experiments were carried out in triplicates.

### 2.2. Fermentation Conditions

The isolated strain was grown in a 2 L fermentor under optimized fermentation conditions. The optimized fermentation minimal medium consists of (g/L) 3% TF, NaCl, 0.4; NH_4_Cl, 0.005; K_2_HPO_4_, 1.25; KH_2_PO_4_, 0.3; and the trace element solution of 1mL containing (g/L) MgSO_4_,0.49; FeSO_4_, 0.055; CoCl_2_, 0.028; MnCl_2_, 0.019; CaCl_2_, 0.147; and NH_4_Mo7O24, 0.123, with pH 8. The media was autoclaved at 120°C at 15 psi for 15 min and fermentation was carried out by seeding 3% of 36 hour inoculums and incubating on a rotary shaker under 200 rpm at 30°C for 48 hours. All the experiments were carried out in duplicates and repeated thrice.

### 2.3. Extraction and Purification of the Pigmented Metabolite (Prodigiosin)

Extraction was carried out according to Heinemann et al. [6]. Prodigiosin was visualized as a fluorescence spot under UV light with Rf value of 0.9 to 0.95. The pigment was purified by column chromatography using silica gel (mesh size 80–100) as the solid matrix for separation of the noncolored impurity from the pigment [7].

### 2.4. Antimicrobial Activity

Antimicrobial activity was determined using the agar well diffusion assay [8]. Antifungal activity of the crude extract was determined by using the standard method CLSI M38-A (formerly NCCLS). The bacterial and fungal pathogenic strains were obtained from Microbial Type Culture Collection (MTCC, Chandigarh, India) and ATCC. Antimicrobial activity were evaluated against bacterial strains namely *Escherichia coli* (MTCC 2939), *Salmonella *typhi (MTCC 98), *Proteus vulgaris *(MTCC 1771), *Pseudomonas aeruginosa *(MTCC 1688), Staphylococus* aureus *(MTCC 96), *Bacillus subtilis *(MTCC 441), and *Klebsiella pneumoniae *(ATCC 10273), and against various fungi, namely, *Aspergillus niger *(MTCC 281), *Trichoderma viridae *(MTCC 167),* Penicillium chrysogenum *(MTCC 160), *Microsporum canis *(MTCC 2820), *Candida albicans *(MTCC 183), *Fusarium moniliforme *(MTCC 156), *Trichophyton rubrum *(MTCC 296)*, Trichophyton mentagrophytes *(ATCC 9533), *Fusarium *oxyzporum (*ATCC* 695), and *Aspergillus flavus (ATCC* 10836).

### 2.5. Cytotoxic Effect of Prodigiosin

*Maintenance of Cell Lines and In Vitro Cytotoxicity Assay (MTT Cell Proliferation Assay and Analysis of DNA Fragmentation).* Human laryngeal cancer (HEp-2), human oral cancer (KB), human cervical adeno carcinoma (HeLa) cell lines, and Vero cells were procured from the National Centre for Cell Sciences (NCCS), Pune, India. Cytotoxicity was measured using an MTT assay and DNA fragmentation studies were carried out according Herrmann et al. [9].

## 3. Results and Discussion

### 3.1. Isolation, Identification, and Growth of the Pigment Producing Bacteria

**Figure 1:**
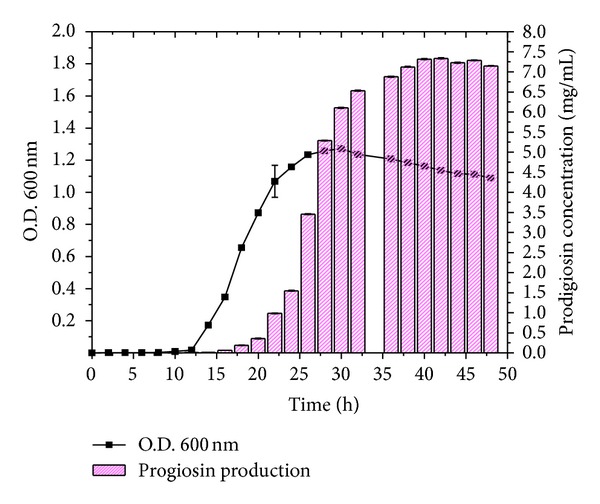
Growth related prodigiosin production in fish gut bacteria.

The acclimatization process with the solid waste (TF) medium enabled the bacterial community inside the gastrointestinal tract of the fish to utilize the proteinaceous TF as a substrate. Prodigiosin production was observed throughout the early log phase to stationary phase. *Serratia marcescens* NPLR1 produced prodigiosin up to 48 hours; however, the maximum peak was observed at the 40th hour. The data revealed that the concentration of prodigiosin increased coordinately with increasing bacterial density (Figure 1) in the 1% TF enriched medium which may be due to the fact that the intracellular concentration of regulator increases to a threshold needed for activation of prodigiosin expression as the cell density increases [10].

### 3.2. Effect of Carbon Sources and Nitrogen Source on Prodigiosin Production

Table 1 depicts the effect of various carbon sources on the production of prodigiosin by the isolated strain at 37°C, pH 7, and 40 h of incubation period. About 1% of bacterial culture (log phase) was used to inoculate the medium and optimize the parameters during the entire period of study. Carbon sources like mannitol, sorbitol, and fructose did not support the growth of the strain and in other carbon sources like starch, arabinose, and dextrose there was almost an inverse relationship between the bacterial growth and prodigiosin yield indicating that carbohydrates were apparently poor nutrient sources and repress prodigiosin production *in S. marcescens*.

The maximum production of prodigiosin was observed with nitrogen sources such as casein and gelatin at 1.5% to 2% concentration, followed by diammonium phosphate, monosodium glutamate, and yeast extract. However, this was not the same with sodium nitrate, ammonium sulfate, and potassium nitrate medium (Table 2) [11].

Similarly, the best carbon and nitrogen sources were combined to determine the maximum prodigiosin production (Table 3). About 4.689 mg/mL of prodigiosin was obtained from the sucrose-gelatin combination (0.5% each) at 37°C in 40 h. However, under the same experimental conditions, the maximum prodigiosin yield was about 8.54 mg/mL in the presence of 1% TF. This shows that the TF contains the essential nutrients for the prodigiosin production.

### 3.3. Effect of Temperature and pH on Prodigiosin Production

**Figure 2:**
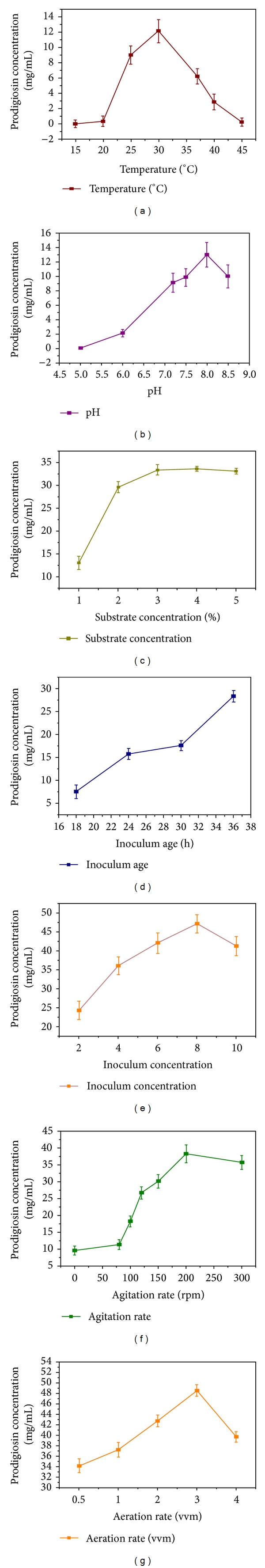
(a) Effect of temperature on prodigiosin production at pH 7 at 40 h. (b) Effect of pH on prodigiosin production at 30°C at 40 h. (c) Effect of substrate concentration on prodigiosin production at temperature of 30°C and pH 8 at 40 h.

Figure 2(a) portrays the effect of temperature on prodigiosin production using tannery fleshing (1%) media at pH 7 after 40 h of incubation period. The trend line exhibited a general increase in pigment production at temperature range 25°C–37°C and the maximum production occurred at 30°C (12 mg/mL) which may be due to inactivation of enzymes involved in prodigiosin synthesis [12].

Figure 2(b) illustrates the effect of pH on prodigiosin production at 30°C after 40 h of incubation. Initially at lower pH, the pigment production was not observed. Pigment production gradually increased after pH 5 and exhibited the peak value at pH 8 (13 mg/mL). This corroborates with earlier findings of the fact that acidic pH below 3.0 or in the alkaline pH above 10.0 prevents pigmentation and an optimum pH range of 7–9 facilitates pigmentation [1].

### 3.4. Effect of Substrate Concentration and Different Media on Prodigiosin Production

3% substrate concentration was found to be sufficient for maximum prodigiosin production (33 mg/mL) (Figure 2(c)). Prodigiosin production noticed in sesame seed broth and peptone glycerol broth was 2.51 mg/mL and 0.674 mg/mL at 30°C and 37°C, respectively. It was observed that the TF containing medium yielded maximum prodigiosin (Table 4). While limited literature is available in support of proteinaceous substrate TF involved in prodigiosin production, some observations do support a role of amino acids in prodigiosin production [5].

### 3.5. Effect of Inoculums Age and Size on Prodigiosin Production

Inoculum age plays a vital role in induction and production of secondary metabolites as the secondary metabolites are induced in the late stationary phase of the bacterial growth.  Figure 2(d) shows the effect of inoculums age on prodigiosin concentration in the fermentation medium under constant experimental conditions. Inoculation of younger cells, for example, 18 h culture produced only 7.5 mg/mL of prodigiosin (at 30°C, pH 8, 3% substrate concentration) since induction, occurred only at the late stationary growth phase in the fermentation medium whereas the older cells of 30 to 36 h culture at 2% cell concentration under the same experimental conditions produced 17.36 to 27.97 mg/mL of prodigiosin as inoculation of already induced aged cells efficiently utilized the fermentation medium (TF) for the biosynthesis of pigments (Figure 2(d)). A subsequent investigation revealed that the size of the inoculums is directly proportional to the fermentation product as it speeds up the reaction by reducing the generation time. As shown in Figure 2(e), lower concentration (1% to 2%) of cells produced low concentration of prodigiosin (25 and 30 mg/mL, resp.) whereas the higher concentration of inoculums (8%) produced higher concentration of prodigiosin (47 mg/mL). Further increase of the inoculum concentration (10%) reduced the prodigiosin production indicating the optimum concentration to be 8%.

### 3.6. Effect of Agitation and Aeration Rates on Prodigiosin Production

Dissolved oxygen and mixing up nutrients are very important in fermentation processes as it plays a key role in nutrient utilization and product formation. Figures 2(f) and 2(g) depict the effect of agitation and aeration rates, respectively, on prodigiosin concentration at 30°C and pH 8 in 40 h of incubation with 8% of 36 h old culture in 3% TF containing medium. These data suggest agitation (200 rpm) and aeration rates (3 vvm) have profound influence on prodigiosin production. The experiment proves that shaking condition with high rate of oxygen transfer was more preferable for pigment production than the static condition. Aeration below or above the optimum level has not facilitated the pigmentation and the obtained results are in agreement with the earlier statement that prodigiosin production is maintained only with fermentors supplied with both aeration and agitation [10].

### 3.7. Instrumental Analysis of the Pigmented Secondary Metabolite

#### 3.7.1. Nuclear Magnetic Resonance Spectroscopy of Pink Pigmented Prodigiosin

**Figure 3:**
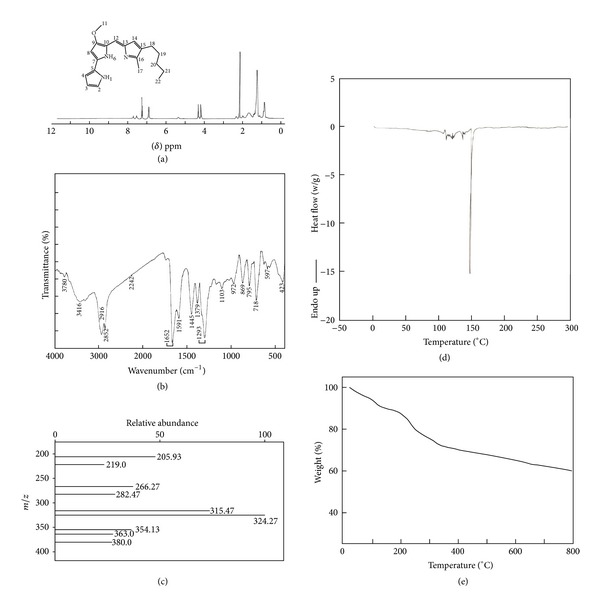
(a) Nuclear magnetic resonance spectrum of purified prodigiosin. (b) Fourier transform infra red spectrum of purified prodigiosin. (c) Electrospray Ionisation-mass spectrometry analysis of purified prodigiosin. (d) Differential scanning calorimetric analysis of purified prodigiosin. (e) Thermo gravimetric analysis of purified prodigiosin.

^1^H-NMR spectrum of prodigiosin represents the peaks corresponding to chemical shifts at *δ*7.23 ppm, *δ*6.95 ppm, *δ*4.01 ppm, *δ*2.17 ppm, *δ*1.28 ppm, and *δ*0.87 ppm assigned to the carbon atoms C2,C12, C11, C18, C21, and C22 based on the structure of prodigiosin presented inset of Figure 3(a).

#### 3.7.2. Fourier Transform Infrared Spectrum of Pigmented Metabolite

From the spectrum obtained, red pigmented prodigiosin showed a broad envelope around 3600–3300 cm^−1^ centered at 3416 cm^−1^ attributed to the –N–H stretch. The peaks at 2916 and 2852 cm^−1^ are due to asymmetrical and symmetrical stretching of methylene groups. The peaks at 1652 cm^−1^ and 1445 cm^−1^ are due to the presence of –NH and methyl groups. The visible peak at 1379 cm^−1^ is due to the presence of C–O group in prodigiosin. The peaks around 1293 cm^−1^ and 718 cm^−1^ are attributed to carbon-carbon double bond (Figure 3(b)).

#### 3.7.3. ElectroSpray Ionisation Mass Spectrum of Prodigiosin

The mass spectrum shows molecular ion peak M + H of prodigiosin at m/z 324 (prodigiosin has molecular weight of 323). Cleavage of –CH_3_CH_2_CH_2_ is reflected at m/z 282. The most important ion peaks of prodigiosin are at m/z 266 which is due to cleavage of a bond beta and meta stable ion peak at m/z 219. The ion peaks at m/z 354, m/z 363, and m/z 380 can be the analogues of prodigiosin (Figure 3(c)).

#### 3.7.4. Thermogravimetric and Differential Scanning Calorimetry (DSC) Analysis

DSC measurements were carried out to assess the thermal properties of prodigiosin. The onset temperature peak was observed at 148°C and a linear melting peak was observed at 137°C (Figure 3(d)).

TGA of prodigiosin (Figure 3(e)) reflects 8.49% weight loss at 122.15°C due to elimination of moisture. TGA records a weight loss of 18.68% in the temperature ranges 122.15 to 326.1°C due to the destabilization of bonds present in the prodigiosin and the decomposition continues up to 80°C (41.18%).

Further, the antimicrobial and cytotoxic potential of the compound obtained was evaluated for its potential medical applications.

### 3.8. Antimicrobial Activity of Prodigiosin

**Figure 4:**
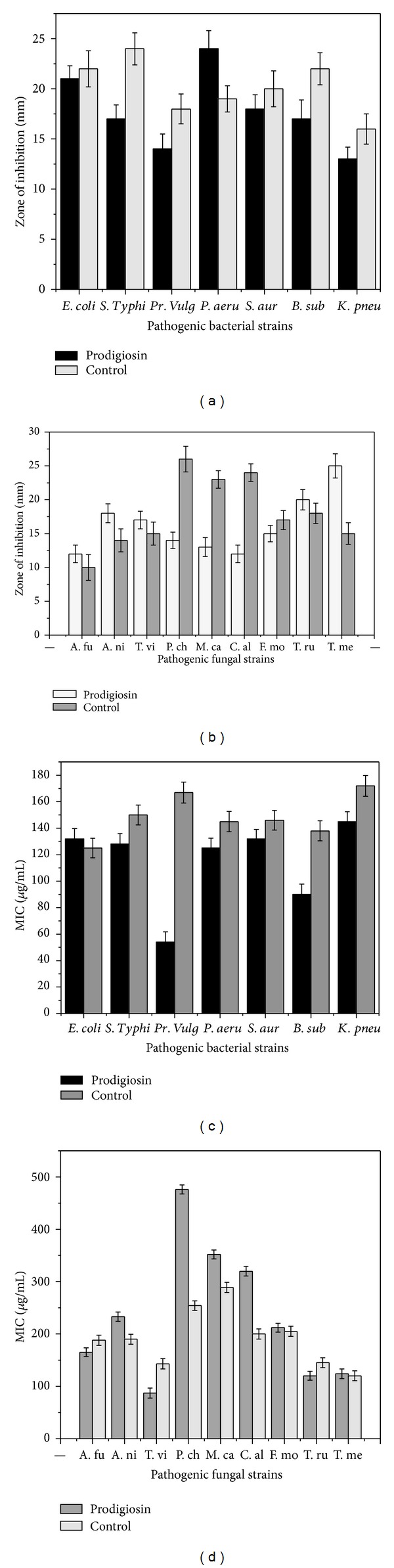
(a) Antibacterial activity of purified prodigiosin. (b) Antifungal activity of purified prodigiosin. (c) Minimum inhibitory concentration of purified prodigiosin against bacterial pathogens. (d) Minimum inhibitory concentration of purified prodigiosin against fungal pathogens.

The bacterial (Figure 4(a)) and fungal (Figure 4(b)) pathogens which exhibited maximum zone of inhibition against prodigiosin were studied in detail. *Pseudomonas aeruginosa* and *E.coli* were more susceptible to prodigiosin. *Klebsiella pneumonia* was the least susceptible bacterial species. The prodigiosin exhibited marked antifungal activity against *Aspergillus niger, Trichoderma viridae, *and *Trichophyton rubrum* better that control (Amphotericin B). However maximum activity was seen in *Trichophyton mentagrophytes. *As shown in Figures 4(c) and 4(d), the secondary metabolite of *S. marcescens,* prodigiosin exhibited concentration dependant inhibition of the bacterial and fungal pathogens. The MIC value differs for the pathogenic strains. Among the pathogenic bacteria *Pseudomonas aeruginosa *was most susceptible at the lowest concentration followed by *Salmonella *typhi.

**Figure 5:**
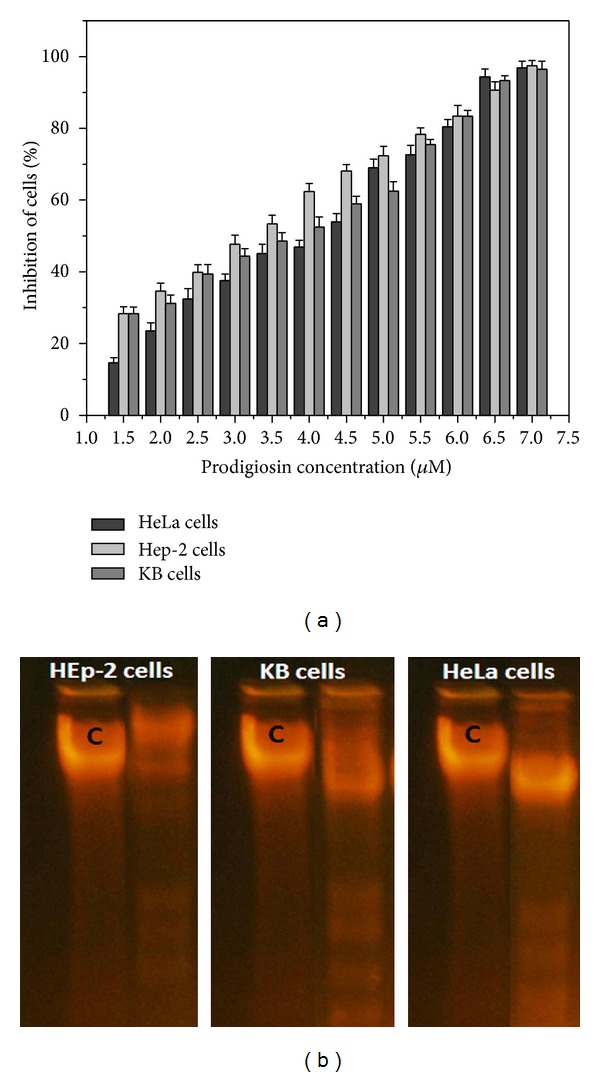
(a) Cytotoxic activity of the purified prodigiosin produced by *Serratia marcescens* against human oral cancer (KB), human cervical adeno carcinoma (HeLa), and human laryngeal cancer (HEp2) cell lines. (b) DNA fragmentation studies by prodigiosin. HEp-2 cells, KB cells, and HeLa cells were treated with IC_50_ concentration of prodigiosin for 48 h. The untreated cell served as control (C). The fragmented DNA was analyzed by agarose gel electrophoresis.

Prodigiosin, a tripyrrole pigment, was found to exhibit antiproliferative property against the human oral cancer (KB), human cervical adenocarcinoma (HeLa) cell, and Human laryngeal cancer (HEp2) cell lines (Figure 5(a)). The relation between concentration of the pigmented metabolite and the cytotoxic effect on the cancer cells was determined to be very strong. However, IC_50_ concentration was cell line specific (HeLa cells: 4.3 *μ*M, HEp2: 5.2 *μ*M, and KB cells: 4.8 *μ*M) and remains nontoxic up to the concentration of 25 *μ*M on normal Vero cells suggesting that cancerous cells are more susceptible to the prodigiosin at lower concentration. Cancerous cells exposed to higher concentration exhibited severe damage in DNA profile and the apoptosis of the cells was further evidenced through DNA fragmentation studies (Figure 5(b)). DNA fragmentation analysis reveals a unique ladder composed of nucleotide fragments at an interval of 180–200 base pairs which are produced by apoptic cells [13]. Similar results were obtained earlier in B-cell chronic lymphocytic leukaemia samples [14], HL-60 cells, and HGT-1 [15]. These results suggest that prodigiosin produced by *Serratia marcescens* utilizing tannery fleshing as a sole carbon and nitrogen sources exhibits potent antitumor activity.

## 4. Conclusion

The extent to which tannery fleshing grown microorganisms become attenuated to produce prodigiosin is reminiscent of the fact that this novel source can be utilized as substrate to enhance the production of the multifaceted metabolite. However, significant improvements would be required to make this approach feasible for larger scale investigations which might prove to be a more fruitful and efficient alternative to existing technologies.

## Figures and Tables

**Table 1:** Effect of different carbon sources as additive on prodigiosin production by *Serratia marcescens* NPLR1 at 37°C and pH 7.

Prodigiosin (mg/L)				
Concentration of carbon sources (w/v)				
Type of carbon sources	0.5%	1%	1.5%	2%
Xylose	0.112	0.165	0.287	0.367
Starch	BDL	BDL	BDL	BDL
Glycerol	0.145	0.576	0.582	0.672
Maltose	1.24	1.58	1.67	1.83
Arabinose	BDL	BDL	BDL	BDL
Galactose	0.138	0.187	0.253	0.276
Sorbitol	NG	NG	NG	NG
Dextrose	BDL	BDL	BDL	BDL
Fructose	NG	NG	NG	NG
Mannitol	NG	NG	NG	NG
Sucrose	1.31	1.65	1.32	1.75

BDL: below detectable limit; NG: no growth.

**Table 2:** Effect of nitrogen sources as additive on prodigiosin production by *Serratia marcescens* NPLR1 at 37°C and pH 7.

Prodigiosin (mg/mL)				
Concentration of nitrogen sources (w/v)				
Nitrogen sources	0.5%	1%	1.5%	2%
Casein	1.65	1.89	2.46	2.97
Gelatin	1.98	2.34	2.67	2.89
Yeast extract	0.156	0.235	0.924	1.276
Peptone	0.169	0.547	1.25	1.62
Tryptone	0.025	0.156	0.324	0.367
Sodium nitrate	BDL	BDL	BDL	BDL
Urea	0.012	0.036	0.089	0.124
Ammonium sulfate	BDL	BDL	BDL	BDL
Ammonium nitrate	0.037	0.068	0.152	0.184
Potassium nitrate	BDL	BDL	BDL	BDL
Monosodium glutamate	0.856	1.23	1.67	1.66
Diammonium phosphate	0.624	0.951	1.35	1.87
Glycine	0.321	0.576	0.864	1.26

BDL: below detectable limit.

**Table 3:** Effect of different combinations of carbon and nitrogen sources as additive on prodigiosin production by *Serratia marcescens* NPLR1 at 37°C and pH 7.

Carbon/nitrogen source combination (1 : 1)	Prodigiosin, mg/mL
Maltose : casein	2.354
Maltose : gelatin	3.641
Maltose : monosodium glutamate	2.76
Maltose : diammonium phosphate	2.34
Sucrose : casein	3.12
Sucrose : gelatin	4.689
Sucrose : monosodium glutamate	3.671
Sucrose : diammonium phosphate	3.12
TF : TF	8.54

**Table 4:** Effect of different broth mediums and temperatures on prodigiosin production by *Serratia marcescens* NPLR1 at pH 7.

Type of broth medium	Prodigiosin (mg/mL) production at 37°C	Prodigiosin (mg/mL) production at 30°C	Prodigiosin (mg/mL) production at 28°C
Nutrient broth	0.021	0.381	0.147
Luria Bertani broth	0.058	0.162	0.086
Peptone glycerol	0.674	0.034	0.153
Potato dextrose	0.005	0.025	0.008
Maltose broth	0.036	0.135	0.076
Sesame seed broth	0.672	2.51	0.945
